# Renal sympathetic denervation inhibites the development of left ventricular mechanical dyssynchrony during the progression of heart failure in dogs

**DOI:** 10.1186/1476-7120-12-47

**Published:** 2014-11-22

**Authors:** Wei Hu, Qing-yan Zhao, Sheng-bo Yu, Bin Sun, Liao Chen, Sheng Cao, Rui-qiang Guo

**Affiliations:** Department of Ultrasound, Renmin Hospital of Wuhan University, Jiefang Road 238#, Wuchang District, Wuhan, 430060 China; Department of Cardiovascular Medicine, Renmin Hospital of Wuhan University, Jiefang Road 238#, Wuchang District, Wuhan, 430060 China; Cardiovascular Research Institute of Hubei University of Medicine, Dongfeng General Hospital, Hubei, 442000 China

**Keywords:** Renal nerve ablation, Sympathetic nervous system, Heart failure, Ventricular dyssynchrony

## Abstract

**Background:**

The purpose of this study was to investigate whether transcatheter renal sympathetic denervation (RSD) interfere with the development of left ventricular (LV) mechanical dyssynchrony during the progression of heart failure (HF).

**Methods:**

Nineteen beagles were randomly divided into sham-operated group (six dogs), control group (seven dogs), and RSD group (six dogs). Sham-operated group were implanted with pacemakers without pacing; Control group were implanted with pacemakers and underwent 3 weeks of rapid right ventricular pacing; and RSD group underwent catheter-based RSD bilaterally and were simultaneously implanted with pacemakers. Both LV strain and LV dyssynchrony were analyzed via 2D speckle-tracking strain echocardiography to evaluate LV function. Longitudinal dyssynchrony was determined as the standard deviation for time-to-peak speckle-tracking strain on apical 4- and 2-chamber views. Radial and circumferential dyssynchrony was determined as the standard deviation for time-to-peak speckle-tracking strain in mid- and base-LV short-axis views. Each myocardial function was also evaluated by averaging the peak systolic strains. LV systolic pressure (LVSP) and LV end-diastolic pressure (LVEDP) were measured. The LV interstitial fibrosis was determined by histological analysis. Plasma angiotensin II (Ang II), aldosterone and norepinephrine (NE) levels were also measured.

**Results:**

After 3 weeks, all of the dogs in both the control and RSD groups showed greater LV end-diastolic volume compared with the sham-operated group; however, the dogs in the RSD group had a higher LV ejection fraction (LVEF) than the dogs in the control group (p < 0.001). The LV systolic strains were higher in the RSD group than in the control group (p < 0.001 for longitudinal, circumferential and radial strain, respectively). The levels of LV dyssynchrony were lower in the RSD group than in the control group (p < 0.001 for longitudinal, circumferential and radial dyssynchrony, respectively). Compared with dogs with control alone, RSD dogs had lower LV end-diastolic pressures and less fibrous tissue. The levels of plasma Ang II, aldosterone and NE were lower in the RSD group than in the control group.

**Conclusions:**

RSD inhibites the development of left ventricular mechanical dyssynchrony during the progression of heart failure in dogs.

## Background

Heart failure (HF) is a major cause of morbidity and mortality, and the epidemic of HF is an important public health issue facing the health care system. Different pathophysiologic mechanisms have been linked to the development and propagation of HF. In cases of reduced cardiac function, several compensation pathways are activated to preserve cardiovascular homeostasis. One of these mechanisms, which plays an essential role in patients with HF, is governed by the neurohormonal system, which consists of the sympathetic nervous system (SNS) and the renin-angiotensin-aldosterone system (RAAS) [1, 2]. However, chronic activation of these neurohormonal signals has deleterious effects on cardiac structure and performance, leading to cardiac decompensation and heart failure progression.

It was recently shown that left ventricular dyssynchrony strongly correlates with the progression of HF [3, 4]. Dyssynchrony in the setting of HF is characterized by nonsynchronous, abnormal electrical activation and an abnormal contraction sequence [5]. More importantly, previous studies have demonstrated that mechanical dyssynchrony is strongly influenced by neurohormonal activity, hemodynamic changes, LV heterogeneity, and myocardial fibrosis [6–8].

The renal sympathetic nervous system has been identified as a major contributor to the complex pathophysiology of both hypertension and HF in humans [9, 10]. Animal studies have suggested that renal sympathetic denervation (RSD) attenuates ventricular and electrophysiological remodeling in animals with heart failure [11–13]. Catheter-based RSD was recently introduced as a technique that protects against heart failure via denervation of efferent and afferent renal sympathetic nerve fibers [14–16]. Following RSD, the activation of both the SNS and RAAS is inhibited [17, 18]. Other studies have demonstrated that treatment with angiotensin-converting enzyme (ACE) inhibitors diminish LV dyssynchrony during the progression of pacing-induced HF, and beta-blocker therapy stimulates increases in local contractility and decreases in intraventricular dyssynchrony [6, 19]. However, the relationship between RSD and LV dyssynchrony remains unknown. Accordingly, the purpose of the present study was to test the hypothesis that RSD attenuates the impairment of LV mechanical dyssynchrony during the progression of HF in dogs.

In recent years, various imaging techniques have been tested to determine their ability to quantify LV dyssynchrony, including magnetic resonance imaging, nuclear imaging, and echocardiography [20–22]. The techniques used most often have included echocardiography using tissue Doppler imaging (TDI) and speckle tracking strain analysis, which measures peak times in different ventricular segments [22, 23]. Furthermore, speckle tracking strain analysis is based on grayscale 2-dimensional images, which allow for the assessment of myocardial deformation in 2 dimensions, including longitudinal strain (LS), which represents myocardial shortening on the long-axis plane; circumferential strain (CS), which represents myocardial shortening on the short-axis plane; and radial strain (RS), which represents myocardial thickening also on the short-axis plane [24].

## Materials and methods

### Animal model

The study protocol was approved by the Ethical Committee of the Wuhan University School of Medicine, and all animal handing was performed in accordance with the Wuhan Directive for Animal Research and the current Guidelines for the Care and Use of Laboratory Animals published by the National Institutes of Health (NIH publication no.85-23, revised 1996). Nineteen beagles of both sexes and approximately one year of age, weighing 13.4 ± 2.1 kg, were divided into the following three groups: 1) the sham-operated group (n = 6); 2), the control group (n = 7); and 3) the RSD group (n = 6). An intramuscular injection of 25 mg/kg of ketamine sulphate was administered to all the dogs. The dogs were then premedicated with pentobarbital sodium (30 mg/kg IV; additional doses of 4 mg/kg were administered when required throughout the experiment), intubated, and ventilated using a respirator with room air supplemented with oxygen (MAO01746; Harvard Apparatus, Holliston, MA, USA). Continuous ECG monitoring was carried out using leads I, II and III.

The pacemakers (Shanghai Fudan University, China) were implanted in a subcutaneous pocket and were attached to a pacing lead (1646 T, St. Jude Medical, Inc, USA) at the right ventricular apex (RVA), under fluoroscopic visualization via the right external jugular vein. In the sham-operated group, when the surgery was completed, the dogs were allowed to recover for 3 weeks without pacing. In the control group, after pacemaker implantation, the dogs were allowed to recover for 3 days, and subsequently underwent rapid ventricular pacing at 240 beats per minute (bpm) for 3 weeks. The dogs in the RSD group underwent double renal artery ablation prior to ventricular rapid pacing. A tailor-made quadrupole radiofrequency ablation catheter was inserted into each renal artery via femoral artery under fluoroscopy (when the quadrupole ablation catheter was introduced into each renal artery, each of the four tips was expanded automatically and attached to the wall of the artery). We applied radiofrequency ablations of 6 W or less and lasting up to 90 sec within each renal artery. The catheter system monitored tip temperature and impedance, altering radiofrequency energy delivery in response to a predetermined algorithm. In the sham-operated group and control group dogs, the ablation catheter was inserted into each renal artery without ablation. In the RSD group, the pacemakers were implanted following ablation, and the dogs recovered for 3 days and underwent pacing for 3 weeks.

### Hemodynamic measurements

An 8 F sheath was inserted percutaneously in the right femoral artery, through which a 5 F pigtail catheter (Terumo) was introduced into the left ventricle through the arterial sheath to detect LV systolic pressure (LVSP) and LV end-diastolic pressure (LVEDP). The pressure signals were continuously recorded and digitalized using Lead 2000 multi-channel physiological signal recorder (Sichuan Jinjiang Electronic Science and Technology Co., Ltd, Sichuan, China).

### Neurohormonal assays

After three weeks, 4 ml of venous blood was collected in EDTA vacutainers and was centrifuged at 2,310 g for 10 min at 4°C (Avanti J-E; Beckman Coulter, Brea, CA, USA). The serum was separated into microtubes and was stored at -80°C until used for analysis. Plasma angiotensin II (Canis Ang II Elisa Kit, Nanjing Jiancheng Bioengineering Institute) and aldosterone (Canis Aldosterone Elisa Kit, Nanjing Jiancheng Bioengineering Institute) concentrations were both measured using ELISA. Plasma norepinephrine (NE) concentrations were measured using high-performance liquid chromatography [25, 26].

### Histological evaluation

At the completion of the protocol, the animals were euthanized and the hearts were quickly excised. The anterior wall of the left ventricle was dissected from the heart and was immediately stored at -80°C. Masson’s Trichrome staining was used to identify increased concentrations of interstitial fibrosis. Connective tissue was differentiated on the basis of its color, and the levels were expressed as percentages of reference tissue areas (Ti-S; Nikon, Tokyo, Japan). Blood vessels and perivascular interstitial cells were excluded from connective tissue quantification. Ventricular interstitial collagen volume fractions were determined by quantitative morphometry using an image analyzer (IPP 6.0; Media Cybernetics, GA, USA).

### Conventional echocardiography

All of the dogs underwent complete transthoracic echocardiographic studies with 2-D, color flow and spectral Doppler using a GE Vivid E9 system (GE Vingmed Ultrasound As, Horten, Norway). Pulse wave Doppler examinations of peak velocities of mitral inflow E and early diastolic myocardial septal annulus movement velocities (E’) were also recorded. LV end-diastolic volumes (LVEDV) and end-systolic volumes (LVESV) were calculated from apical views, using the modified Simpson method [27]. LV stroke volume (SV) was calculated from the difference between LV end-diastolic and end-systolic volumes. LV ejection fraction (EF) was calculated by dividing the stroke volume by the LV end-diastolic volume [28].

### 2-D speckle tracking imaging of the left ventricle

**Figure 1:**
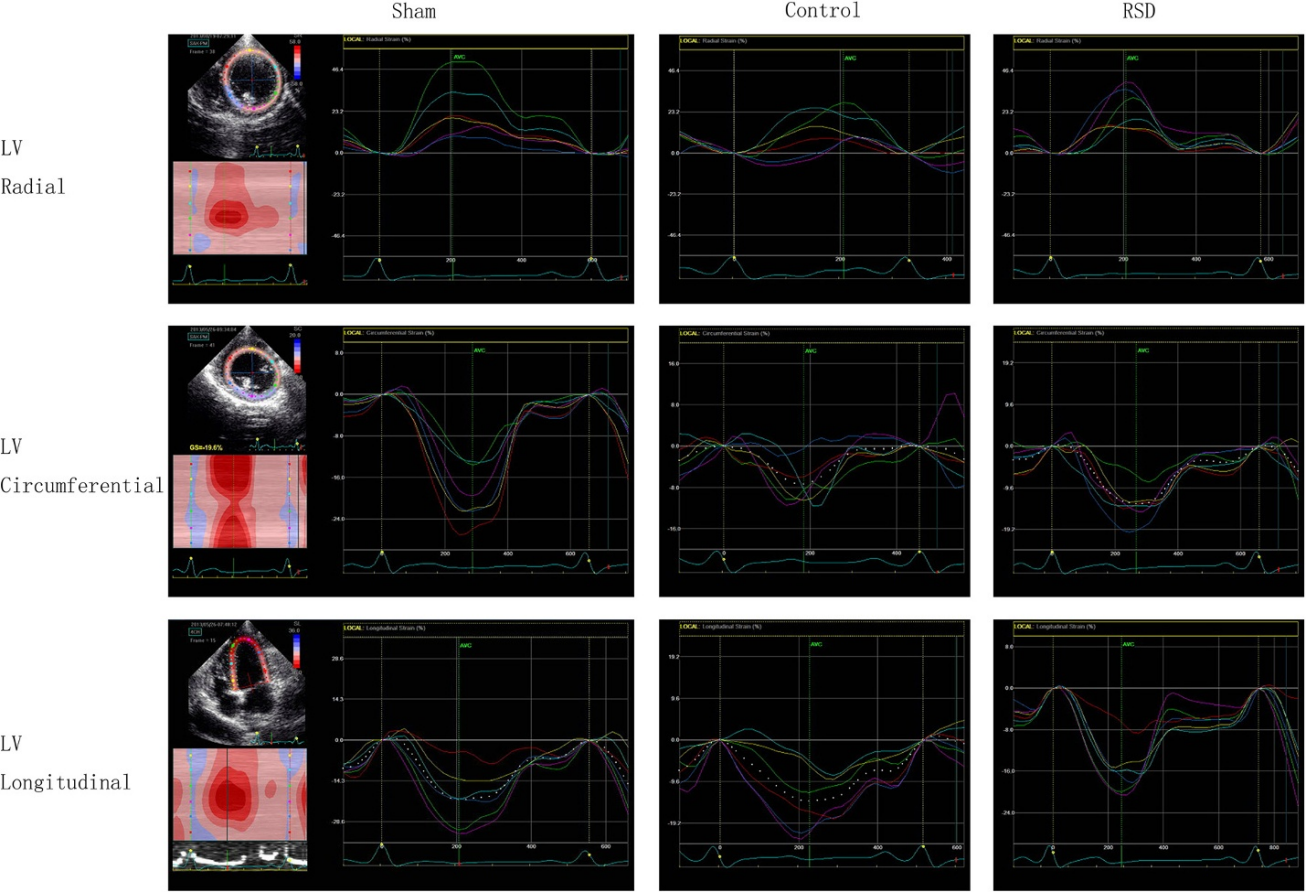
**LV dyssynchrony in three directions (radial, circumferential and longitudinal) in the sham-operated group (sham), the pacing-induced heart failure group (control), and the group treated with renal sympathetic denervation and rapid ventricular pacing (RSD).** LV mechanical dyssynchrony was assessed using the standard deviation of the time from onset of the QRS interval to peak strain.

Left ventricular strain and dyssynchrony were analyzed via two-dimensional speckle tracking echocardiography, using an ECHOPAC workstation as previously described [29]. Routine two-dimensional images were obtained via mid- and base-LV short-axis views and apical four- and two-chamber views at rates of 40–60 frames/s. The speckles of interest were followed throughout the entire cardiac cycle, and myocardial longitudinal, radial and circumferential deformation was each calculated automatically. Longitudinal strain of the LV (LVLS) was calculated as the average peak longitudinal strain across 12 segments of apical four- and two-chamber views. LV circumferential strain (LVCS) and LV radial strain (LVRS) were obtained from 12 segments of mid- and base-LV short-axis views. Mechanical dyssynchrony was assessed using the standard deviations of the time from onset of the QRS interval to the peak longitudinal strain of the LV (LV-Tls-12SD), the peak radial strain of the LV (LV-Trs-12SD) and the peak circumferential strain of the LV (LV-Tcs-12SD) (Figure 1).

The echocardiography was performed after turning off the pacemaker. Digitally stored echocardiographic data were analyzed by an experienced sonographer, and all of the measurements represent an average of 3 to 5 consecutive cycles.

### Statistical analysis

The data are presented as the means ± standard deviations. For between-group changes, ANOVA with Newman-Keuls tests was used to compare the means of continuous variables among multiple groups. In cases in which a significant difference was found, further analysis was undertaken using the Tukey-Kramer test. All of the statistical tests were two sided, and a probability of p < 0.05 was required for statistical significance (version 19.0 SPSS).

## Results

### Conventional echocardiography

Conventional echocardiographic images of all of the subjects were successfully obtained and analyzed, and all of the conventional echocardiographic characteristics of this study are summarized in Table 1. The baseline measurements (LVEDV, LVESV, LVEF, HR, LVEDP and LVSP) among these three groups did not show significant difference (data not shown). After 3 weeks, the dogs in the RSD group demonstrated superior heart function compared to the dogs in the control group, as evidenced by lower LVEDV and LVESV measurements, higher LVSV and LVEF measurements, a lower mitral E/E’ ratio and a higher systolic strain.

**Table 1:** **Routine echocardiographic and hemodynamic characteristics**

	Sham	Control	RSD	p
E (cm/s)	86.83 ± 2.32	72.00 ± 2.16*	77.50 ± 1.64*^†^	0.000
E’ (cm/s)	9.37 ± 0.52	4.96 ± 0.41*	6.39 ± 0.32*^†^	0.000
E/E’	9.29 ± 0.40	14.58 ± 0.84*	12.16 ± 0.68*^†^	0.000
LVEDV (ml)	27.50 ± 1.05	35.86 ± 1.07*	31.50 ± 1.05*^†^	0.000
LVESV (ml)	9.17 ± 0.41	22.57 ± 0.98*	16.33 ± 0.52*^†^	0.000
LVSV (ml)	18.33 ± 0.82	13.29 ± 0.49*	15.17 ± 1.17*^†^	0.000
LVEF (%)	66.67 ± 1.08	37.07 ± 1.34*	48.10 ± 2.35*^†^	0.000
HR (beats/min)	110.17 ± 5.1	109.57 ± 3.1	106.17 ± 3.6	0.199
QRS duration (ms)	66.83 ± 2.86	67.29 ± 3.77	69.17 ± 2.40	0.404
LVEDP (mmHg)	5.5 ± 1.05	24.71 ± 2.14*	12.50 ± 1.05*^†^	0.000
LVSP (mmHg)	153.17 ± 4.07	124.71 ± 4.68*	130.83 ± 6.79*	0.000

Data are expressed as the means ± standard deviations; *p < 0.05 vs. the sham-operated group; ^†^p < 0.05 vs. the control group;E: mitral early diastolic filling velocity; E’: mitral early diastolic annular velocity; LVEDV: left ventricular end-diastolic volume; LVESV: left ventricular end-systolic volume; LVSV: left ventricular stroke volume; EF: ejection fraction; HR: heart rate; LVEDP: left ventricular end-diastolic pressure; LVSP: left ventricular systolic pressure.

### LV dyssynchrony evaluation

**Figure 2:**
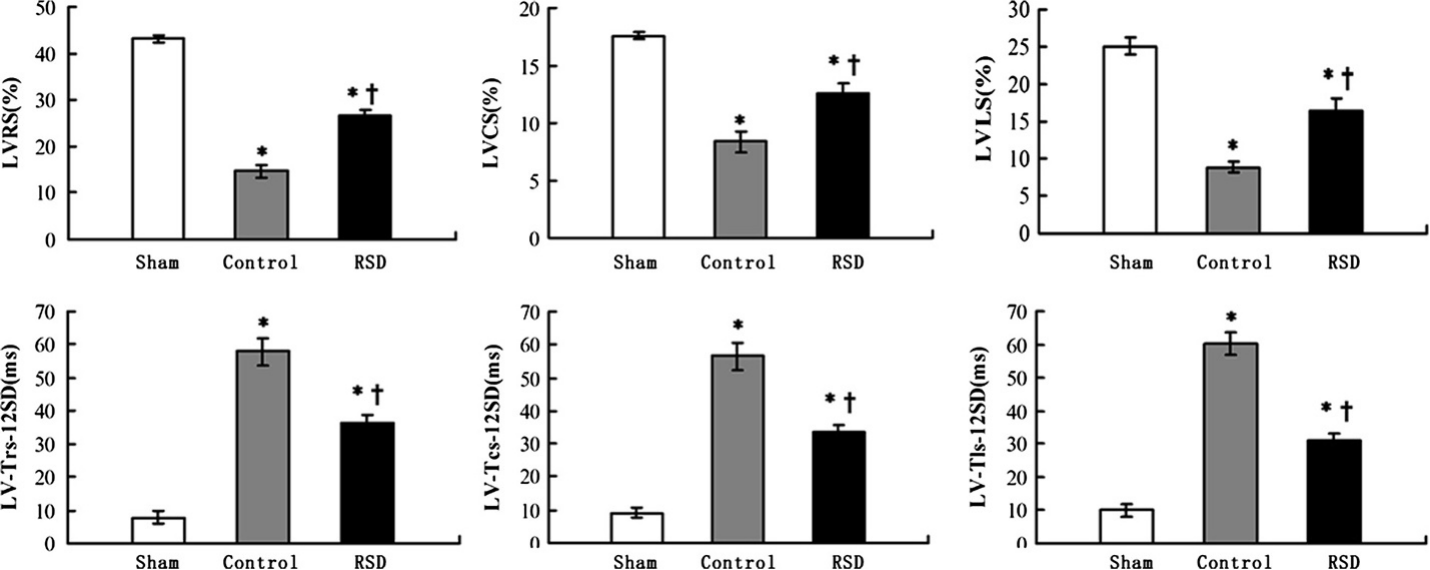
**Left ventricular strain and dyssynchrony in three directions (longitudinal, radial and circumferential) in the sham-operated group (sham), the pacing-induced heart failure group (control), and the group treated with renal sympathetic denervation and rapid ventricular pacing (RSD).** *p < 0.05 vs. the sham-operated group; ^†^p < 0.05 vs. the control group. LV: left ventricular; RS: radial strain; CS: circumferential strain; LS: longitudinal strain; SD: standard deviation.

The peak times to left ventricular systolic strain were not significantly different between the RSD group and the control group (p > 0.05) (Table 2). In contrast, the levels of longitudinal dyssynchrony, radial dyssynchrony and circumferential dyssynchrony in the control group were higher than those in the sham-operated group, the differences were statistically significant (p < 0.001). However, the levels of LV longitudinal, radial and circumferential dyssynchrony in the RSD group were 30.92 ± 2.34 ms, 36.42 ± 2.58 ms and 33.60 ± 1.94 ms, respectively, which are significantly lower than those in the control group (Figure 2).

**Table 2:** **Times to left ventricular peak systolic strain**

	Sham	Control	RSD	p
LV-Tls (ms)	217.58 ± 11.31	238.39 ± 27.03	232.32 ± 14.74	0.182
LV-Tcs (ms)	214.56 ± 22.78	233.29 ± 16.52	223.63 ± 20.00	0.261
LV-Trs (ms)	217.85 ± 8.41	234.42 ± 16.49	226.03 ± 18.63	0.180

Data are expressed as the means ± standard deviations; LV: left ventricular; Tls: time to peak longitudinal strain; Tcs: time to peak circumferential strain; Trs: time to peak radial strain.

### Hemodynamic effects of pacing

After the pacemakers were turned off for 30 min, hemodynamic measurements were obtained. The LV systolic pressure (LVSP) was lower in the RSD group than in the sham-operated group (130.83 ± 6.79 mm Hg vs. 153.17 ± 4.07 mm Hg, p < 0.001) but was not significantly different from that in the control group. The LV end-diastolic pressure (LVEDP) was lower in the RSD group than in the control group (12.50 ± 1.05 mm Hg vs. 24.71 ± 2.14 mm Hg, p < 0.001), but it remained higher than in the sham-operated group (p < 0.001) (Table 1).

### Neurohormonal activity

**Figure 3:**
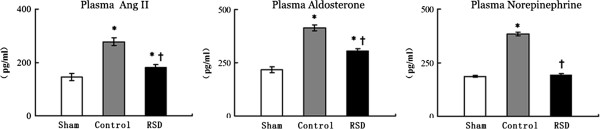
**Plasma angiotensin II, plasma aldosterone and plasma norepinephrine levels in the sham-operated group (sham), the pacing-induced heart failure group (control), and the group treated with renal sympathetic denervation and rapid ventricular pacing (RSD).** *p < 0.05 vs. the sham-operated group; ^†^p < 0.05 vs. the control group.

The plasma angiotensin II (Ang II) levels were lower in the RSD group compared with the control group (p < 0.05), but they remained higher than those in the sham-operated group (p < 0.05). The plasma aldosterone levels were significantly lower in the RSD group compared with the control group, but they remained higher than in the sham-operated group (p < 0.05). The plasma norepinephrine levels were significantly higher in the control group compared to the sham-operated group, although they were slightly attenuated in the RSD group (Figure 3).

### Histological changes

**Figure 4:**
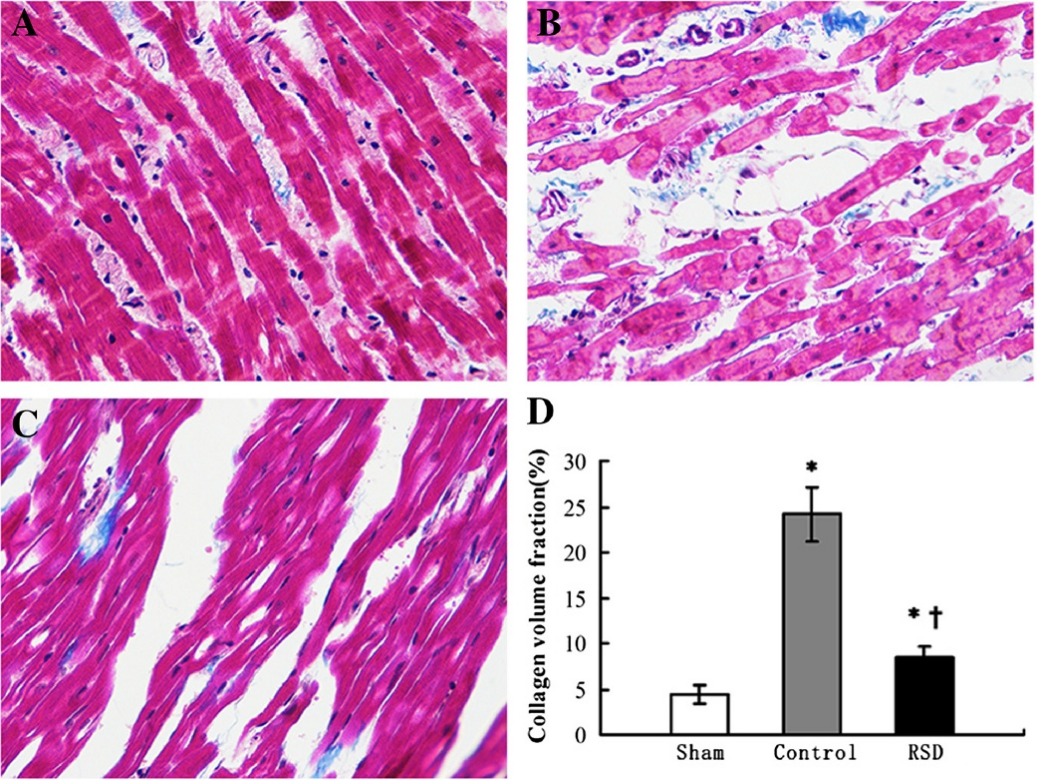
**Histological changes in the left ventricular tissues of the sham-operated group (A), the control group (B) and the RSD group (C).** Summary of changes in the three groups **(D)**. Red areas represent myocytes, and blue areas represent collagen (original magnification: 400). Left ventricular sections in the RSD group had less fibrosis than in the control group. *p < 0.05 vs. the sham-operated group; ^†^p < 0.05 vs. the control group.

Masson’s Trichrome staining was used to evaluate the extent of fibrosis in each tissue section. Left ventricular walls from each of the three groups were composed of red myocardial tissue and blue collagen fibers (Figure 4). Left ventricular images from the control group revealed a large amount of fibrosis (24.2 ± 3.0%), whereas the sham-operated group demonstrated minimal fibrotic tissue (4.4 ± 1.0%). In contrast, the RSD group demonstrated significantly less fibrosis than the control group (8.5 ± 1.1% vs. 24.2 ± 3.0%, p < 0.05) (Figure 4).

## Discussion

The major findings of our study were that RSD attenuated the development of LV dyssynchrony after 3 weeks rapid ventricular pacing and alleviated the activation of neurohormonal signals.

Speckle-tracking echocardiography is an angle-independent technique used to quantify myocardial motion [24, 30]. Previous studies have used this novel method to evaluate LV dyssynchrony [31]. In the present study, both left ventricular EF and systolic function were reduced in the control group, suggesting that this HF model was successful and was consistent with that of a previous study [32]. Furthermore, RSD partially inhibited the impairment of LV systolic function. Jialu Hu et al. found that RSD had both preventive and therapeutic effects on post-myocardial infarction cardiac remodeling and that ejection fraction (EF) was partially protected by RSD [11], consistent with the findings of our study. More importantly, we found that the LV longitudinal, radial and circumferential mechanical dyssynchronies were partially inhibited by RSD.

HF is a clinical syndrome that develops in response to an insult, resulting in a decline in the pumping capacity of the heart. To compensate, neurohumoral mechanisms, such as the SNS and RAAS, are activated [33]. These systems initially compensate for depressed myocardial function, and they preserve cardiovascular homeostasis. However, their long-term activation has deleterious effects on cardiac structure and performance, leading to myocardial remodeling, hemodynamic changes and myocardial fibrosis [34, 35].

LV mechanical dyssynchrony is strongly influenced by hemodynamic changes (e.g., increases in preload and afterload). Min-Seok et al. observed that nitroglycerin improved LV mechanical dyssynchrony by reducing preload. Conversely, the leg-raising maneuver significantly elevated LV mechanical dyssynchrony by increasing preload [36]. The E/E’ ratio, which represents the LV filling pressure, is correlated strongly with LV dyssynchrony in hypertensive patients [37], indicating that preload conditions are closely associated with LV dyssynchrony. In the present study, the RSD group exhibited decreased preload (LV end-diastolic pressure and LV end-diastolic volume) compared to the control group, which might have affected LV dyssynchrony. Miura et al. demonstrated that impaired relaxation with afterload augmentation induced by angiotensin II infusions caused LV mechanical dyssynchrony [7], and Hyo et al. reported that LV dyssynchrony was significantly affected by alterations in LV end-systolic wall stress [38], suggesting that increased afterload might contribute to increased LV dyssynchrony. However, in the present study, the afterload (LV systolic pressure) was reduced in the RSD and control groups compared with the sham-operated group, showing that LV dyssynchrony is affected by many factors.

The relationship between LV mechanical dyssynchrony and myocardial fibrosis has been discussed. Myocardial fibrosis can induce segmental wall asynergy, leading to electrical and mechanical dyssynchrony [39, 40], and dyssynchronous wall motion is more pronounced in patients with healed myocardial infarctions [41]. Furthermore, both the location and the extent of myocardial scarring are determinants of responsiveness to CRT because patients with transmural scars at the location of the LV lead, as well as patients with large areas of myocardial scarring, show lower response rates to CRT compared to patients with minimal scar tissue [42–44]. In the present study, we found that RSD inhibited the increasing of Ang II, aldosterone and norepinephrine, and the impairment of LV fibrosis was sharply alleviated by RSD. So, RSD attenuated the development of the ventricular remodeling induced by long-term rapid ventricular pacing, consistent with the findings of a previous study [39], suggesting that RSD attenuate the development of left ventricular dyssynchrony partly through the suppression of neurohormonal activation in the setting of experimental HF.

## Conclusion

Our study showed that RSD attenuates the development of left ventricular dysfunction and dyssynchrony during the progresses of HF partly by suppressing neurohormonal activation. Our findings suggested that RSD may be a useful therapeutic method for the prevation and treatment of LV dyssynchrony in the setting of HF.

### Study limitations

This study had several limitations. First, two-dimensional speckle tracking has several technical limitations because the accuracy of the strain values depends on the quality of the images and the frame rates. Second, we did not detect interventricular mechanical dyssynchrony. Although interventricular mechanical dyssynchrony is a strong predictor of cardiac function, there is no simple and reliable clinical method of measuring it. Third, the effects of heart rate on mechanical dyssynchrony might have confounded our results. In this study, decreasing trends in heart rate were noted in both the control and RSD groups compared with the sham-operated group, but no significant differences existed among the groups. Fourth, RSD plays an important role in regulating blood pressure, therefore, the protective effect of RSD on cardiac function may be partially attribute to the lowering effect of the blood pressure in this study. But, the changes of blood pressure have not been observed in three groups.

## Abbreviations

RAAS: The renin-angiotensin-aldosterone system
SNS: Sympathetic nervous system
RSD: Renal sympathetic denervation
HF: Heart failure
LV: Left ventricular
LV Tsls-6SD: The standard deviation of time to LV longitudinal peak systolic strain by six segments
AngII: Angiotensin
NE: Norepinephrine
ACE: Angiotensin-converting enzyme
E’: The early diastolic tissue Doppler velocity of the mitral annulus
EDV: End-diastolic volumes
ESV: End-systolic volumes
SV: Stroke volume
EF: Ejection fraction
LS: Longitudinal strain
CS: Circumferential strain
RS: Radial strain
LVSP: LV systolic pressure
LVEDP: LV end-diastolic pressure.
